# End-colostomy parastomal hernia repair: a systematic review on laparoscopic and robotic approaches

**DOI:** 10.1007/s10029-024-03026-8

**Published:** 2024-04-16

**Authors:** G. Sarno, B. Iacone, A. Tedesco, A. Gargiulo, A. Ranieri, A. Giordano, S. Tramontano, U. Bracale

**Affiliations:** General and Emergency Surgery Unit, Department of Medicine, Surgery and Dentistry, “Salerno Medical School”, San Giovanni di Dio e Ruggi d’Aragona University Hospital, Scuola Medica Salernitana, University of Salerno, Faculty of Medicine and Surgery, Campus di Baronissi (SA), “Gaetano Fucito” Facility, Mercato San Severino (SA), Salerno, Italy

**Keywords:** Parastomal hernia, Colostomy, Robotic, Laparoscopic, Mininvasive surgery

## Abstract

**Introduction:**

Parastomal hernia (PSH) is the most common and challenging complication after stoma creation, with an estimated 50% incidence 2 years after the index surgery. Mesh repair is the treatment of choice. Laparoscopic and/or robotic approaches allow acceptable outcomes.

**Materials and methods:**

A systematic literature review from January 2012 to November 2023 was conducted according to the Preferred Reporting Items for Systematic Reviews and Meta-Analyses (PRISMA) statement. Embase, PubMed, and Scopus search were performed to select articles dealing with minimally invasive surgical treatment for PSH after end colostomy.

**Results:**

603 studies were found, and 24 were chosen. When compared to open surgery, laparoscopy showed decreased postoperative complications and recurrence. The main laparoscopic approaches are the keyhole (KH), the Sugarbaker (SB), and the sandwich technique. Continuous improvement in surgery, mesh technology, and surgeons’ expertise led to an amelioration of surgical outcome and recurrence rate after repair. Recent studies showed comparable outcomes for SB and KH techniques, while novel surgical approaches have been proposed in an attempt to further increase the operative and long-term results. Reports on PSH robotic repairs are scarce and describe small series results, suggesting a role of the initial learning curve as a risk factor for complications.

**Conclusion:**

End-colostomy PSH surgical repair still represents a challenge for surgeons. Recent evidence has not shown a significant advantage in postoperative complications and recurrence with a specific repair among SB, KH, and sandwich technique. The paucity of data on robotic surgery does not allow to draw definitive conclusion. Further primary, multicentric, and larger cohort studies are needed.

## Background

Parastomal hernia (PSH) is the most common and challenging complication after stoma creation. This condition, defined as an abnormal protrusion of the abdominal cavity contents through abdominal wall defect created during placement of an enterostomy [1, 2], occur up to nearly 50% of patients [3–5] usually within 2 years after stoma formation [6]. Due to the several issues either in diagnosis than in surgical repair, such kinds of abdominal wall hernias are currently recognized in the field of “complex abdomen”, with complexity defined by patients’ medical history and morbidity, features of the hernia along with previous abdominal surgeries, and attempts at repair. These conditions indicate the need of a highly specialized and tailored surgical approach, but unfortunately even when a repair is performed in a high volume surgical center by an experienced surgeon, postoperative complications as well as hernia recurrence might occur over the follow-up. A consensus on the choice of a specific surgical technique for repair is lacking [7].

The incidence of PSH is particularly high after colostomy formation, whose incidence has been reported as high as in 39% of patients [3, 8, 9]. Yet, the real incidence of this complication may be underestimated because of a wide qualitative, methodological, and follow-up heterogeneity of the various studies considered and can reach higher values [10]. As a matter of fact, the reported incidence of PSH is strongly related to the method of diagnosis, usually by the mean of a clinical examination, and more rarely on an imaging-based study. When diagnosis was achieved by the addition of abdominal computed tomography (CT) scan to the physical examination, the incidence raised up to 78% [11].

Therefore, it must be considered that an enterostomy is in itself, by definition, (although intentional) an incisional hernia, so that some surgeons have stated that PSH is even unavoidable [4, 12], the reason being that accuracy at index surgery is mandatory to avoid PSH onset. Up to 25% of PSH are asymptomatic [9] and can be treated conservatively. Commonly, in asymptomatic cases, to minimize discomfort and further complications, the strategies of choice comprise patients’ education, hernia belts, weight loss, avoidance of heavy lifting [6]. Anyway, a surgical approach is the only available treatment to warrant repair, but the ideal surgical repair for PSH remains undefined. Nevertheless, several indications for surgery have been described over time. A previous attempt to clarify this issue was conducted in 2012 by Hansson et al. [8]. The authors carried out a systematic review analyzing all the available literature over the time span between January 1950 and November 2010 and recommended the use of a mesh in PSH to reduce the risk of recurrence. Although there have been improvement in surgical techniques, the introduction of mesh, and continuous amelioration of mesh technology, surgery for PSH repair is still a challenge for surgeons, mainly because of the need to balance the restoration of the parastomal abdominal wall against stoma malfunction and the upcoming risk of PSH recurrence. If a suture non-mesh repair is no more recommended except in emergency setting because of the excessive risk of recurrence, mesh augmentation accounting for 90% of PSH repairs is the technique of choice, allowing to significantly reduce the risk of recurrence [5, 13]. Minimally invasive repairs, through laparoscopy and more recently by robotic approach [14–17], seem to be the treatment of choice. The objective of this manuscript is to systematically review the current up-to-date evidence concerning minimally invasive techniques focusing on end-colostomy PSH repair in the setting of a laparoscopic or robotic approach.

## Materials and methods

A systematic literature review was conducted following the Preferred Reporting Items for Systematic Reviews and Meta-Analyses (PRISMA) statement [18]. We searched Embase, PubMed, and Scopus to select articles published from January 2012 to November 2023 related to the minimally invasive treatment of PSH after end-colostomy formation. The search algorithm used is shown in Appendix. The articles were independently screened for title and abstract by two reviewers (B.I. and A.T.); subsequently, the full text of the included articles was further analyzed. Any disagreements were resolved through consensus. The exclusion criteria were case reports, reviews, editorials, opinion articles and vignettes, no English written studies, studies with a follow-up shorter than 12 months.

## Results

**Figure 1:**
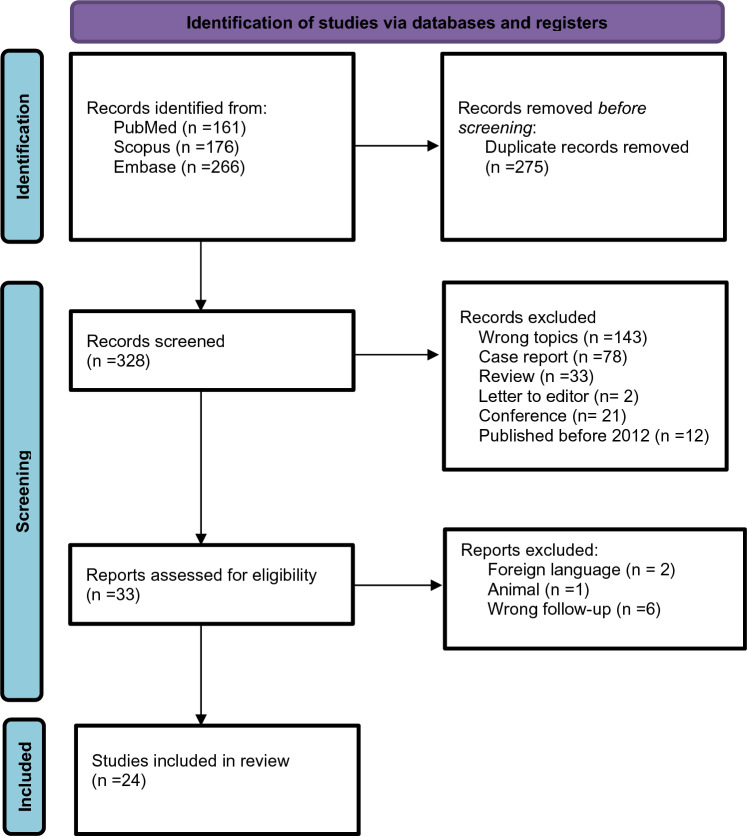
Flow diagram PRISMA

603 studies were found in the literature. Duplicate studies or those that did not meet eligibility criteria were excluded (n = 570). The full texts of 33 studies were evaluated and 9 of them were excluded because they did not align with the inclusion criteria. We selected 24 studies, and data regarding PSH repair after end-colostomy formation were extracted from all included studies (Fig. 1). The details of the included studies are presented in Table 1.

**Table 1 D:** etails of the studies reported in this review

Author	Year	Study design	No. of colostomies	Indication for initial stoma creation	Surgery technique	Stoma type	Recurrent PSH	Type of mesh	Mesh position	Complications	Mean follow-up (months)	Recurrences	Results
Laparoscopic approach													
A. Asif [6]	2012	Retrospective study	16	IBD (30), Cancer (9), Diverticulitis (5), Other (5)	Laparoscopic modified Sugarbaker (14), laparoscopic Keyhole (19), ostomy re-sitings (11), open primary repair (5)	Ileostomy (33) colostomy (16)	19	Gore Dual-Mesh^®^	Intraperitoneal	Ileus (11), wound infection (6), small bowel obstruction (1), pneumonia (1)	7.5–35.8	19	The modified SB technique may offer patients a significant decrease in the risk of recurrence compared with other PH repair techniques with no significant increase in postoperative complication
B. M. E. Hansson [31]	2012	Retrospective multicenter study	55	Colorectal and anal malignancy (43); bladder carcinoma (2); IBD 86); diverticulitis (6); incontinence (3); benign rectal stenosis (1)	Laparoscopic modified Sugarbaker technique	Colostomy (55), ileostomy (4), urostomy (2)	11	Gore-Tex Dual-Mesh Biomaterial^®^	Intraperitoneal	Wound infection (1), postoperative ileus (6), trocar site bleeding (2), mesh infection (1), pneumonia(1), seroma (12)	26	4	Laparoscopic parastomal hernia repair using the Sugarbaker technique with an ePTFE mesh is safe, with recurrence rate of 6.6%. A laparoscopic approach revealed a concomitant incisional hernia in 41% of the patients, which was repaired at the same time
F. J. DeAsis [23]	2015	Retrospective study	23	Unspecified	Laparoscopic modified Sugarbaker (25), Keyhole (18), stoma relocation (13), open repairs (6)	Colostomy (23), ileostomy (39)	23	Gore Dual-Mesh^®^	Intraperitoneal	Hemorrhage (Sugarbaker 1, Keyhole 1, open repairs 1), ileus (Sugarbaker 4, Keyhole 9, Re-siting 3, open repairs 1), mesh infection (Sugarbaker 1), wound infection (Sugarbaker 2, Keyhole 3, Re-siting 2, open repairs 1), small bowel obstruction(Keyhole 2), urinary tract infection (Sugarbaker 1)	Sugarbaker 16.6, Keyhole 19.6, re-siting 16.4, open repairs 27.2	4 (Sugarbaker), 11 (Keyhole), 9 (Re-siting), 2 (open)	A laparoscopic modified SB technique provides decreased rates of recurrence and postoperative complications compared with other approaches
G. Kohler [48]	2015	Retrospective study	120	Oncological (117), non malignant disease (18)	Fascial sutures (25), Onlay mesh (22), Sublay mesh (20), Laparoscopic keyhole (22), Laparoscopic Sugarbaker (4), Laparoscopic sandwich (21), Laparoscopic same-side stoma relocation through funnel mesh (16), Open same-side stoma relocation through funnel mesh (5)	Colostomy (120), Ileostomy (10), Urostomy (5)	31	Vypro or Vypro II, Ultrapro, Parietene,parietex composite, DynaMesh-IPOM, DynaMesh-IPST	Onlay, sublay, and intraperitoneal methods	Bowel injurie (2 laparoscopic), mesh infections (2 open), surgical site infections (8), subcutaneous seromas (2)	54	44	The results achieved by direct suture or the use of incised flat meshes for the repair of PSH were poor with these procedures having unacceptably high recurrence rates. With regard to the latter ostomy revision through three-dimensional funnel-shaped meshes and the laparoscopic sandwich technique showed the best results. Emergency procedures were linked to a dramatic increase in morbidity and mortality
M. Szczepkowski [28]	2015	Prospective single surgeon study	12	Colorectal cancer (9), prostate cancer (1), rectal prolapse (1) rectovaginal fistula (1)	HyPER/SPHR technique (hybrid parastomal endoscopic re-do/Szczepkowski parastomal hernia repair)	Colostomy (12)	0	DynaMesh-IPST^®^	Intraperitoneal	No stoma site infection (SSI) or stoma-related problems were found	13.5	0	Early results show that is has a high patient satisfaction rate and a low number of complications. This novel approach seems to be very promising in terms of the complication rate and recurrence rate
K. Suwa [49]	2016	Retrospective study	16	Rectal cancer (7), Bladder cancer (3), Perforation of sigmoid colon (3), Colon cancer (1), Fournier’s gangrene (1), Perineal Paget’s disease (1)	Laparoscopic modified Sugarbaker	Colostomy (13), ileal conduit (3)	2	Parietex™ Parastomal Mesh (PCO-PM)	Intraperitoneal	Ureteral obstruction (1), enterotomy intraoperative (1)	14.5	0	Sugarbaker’s modified laparoscopic approach to parastomal hernia repair, using the PCO-PM technique, was found to be safe and effective, with no recurrences observed during the average follow-up of 14.5 months and with a low incidence of complications
I. Fischer [27]	2017	Retrospective analysis	47	Unspecified	3D funnel mesh in IPOM technique	Colostomy (47) Ileostomy (2) Urostomy (7)	17	DynaMesh-IPST^®^	Intraperitoneal	Local infections and seroma (4), stoma necrosis (1), stoma retraction (1), bleeding (1), ileus (1), iatrogenic bowel lesion (1)	38	4 (open), 3 (laparoscopic)	Long-term clinical follow-up with standardly integrated CT scan, we did not detect any severe mesh-related complications
G. Kohler [38]	2017	Pilot prospective case series	10	Oncological (12)	Combined laparoscopic and ostomy-opening approach	Colostomy (10), ileostomy (1), urostomy (1)	Unspecified	DynaMesh-IPST	Intraperitoneal	Superficial peristomal wound defect (1)	36	12%	The technique described gives several advantages, such as a minimally invasive hybrid approach creating a real three-dimensional mesh-covered barrier between the trephine and stomal limb and optional shortening of a concomitant prolapse. When needed due to a concomitant incisional hernia, a second flat mesh can be laparoscopically placed in an intraperitoneal position
E. Oma [12]	2018	Retrospective study	79	Colonic malignancy (2), rectal malignancy (41) Anal malignancy (1), Diverticular disease (3), anal fistula (3), IBD (16), adhesive small bowel obstruction (1), obstructed defecation (4), fecal incontinence (7), congenital anomaly (1)	Keyhole (10), Sugarbaker (69)	60 colostomy, 19 ileostomy	5	Parietex™ Composite Parastomal Mesh	Intraperitoneal	Obstruction due to mesh (3), Abscess (1), Adhesions (1), Subcutaneous prolapse (20), Chronic pain (3)	12	1 (keyhole), 6 (Sugarbaker)	This study demonstrated that this mesh material was an excellent choice for parastomal hernia repair performed by experienced surgeons and found low rates of recurrence and chronic pain following parastomal hernia repair using intra- peritoneal reinforcement with a polyester monofilament composite mesh
S. Rege [34]	2018	Retrospective study	12	Carcinoma rectum (12), IBD (1), carcinoma urinary bladder (1)	Modified Sugarbaker	Colostomy (12), ileostomy (1), ileal conduit (1)	Unspecified	Parietex Composite Mesh	Intraperitoneal	No complications as recurrence, seroma, mesh infections or erosions into the stoma	15–85	0	Laparoscopic repair of parastomal hernias with modified mesh placement in the Sugarbaker technique could help reduce mesh-related complications, thereby reducing the risk of hernia recurrence, in addition to the advantages of minimally accessible surgery
Z. Yan [10]	2018	Retrospective study	60	Colorectal and anal malignancy (60), IBD (4), Bladder carcinoma (1)	Modified laparoscopic keyhole parastomal hernia repair with in situ re-ostomy	Ileostomy (4), Colostomy (60), Urostomy (1)	2	Sepramesh™ IP Composite prosthesis or Parietex^®^composite mesh	Intraperitoneal	Seroma (2), ileus (3), intestinal perforation (1)	29 ± 2.1	1	White this technique, patients not only receive the benefits of minimally invasive surgery but also suffer less abdominal trauma, without an extra incision. The recurrence rate was lower than that of other techniques during the follow-up period
S. Olmi [29]	2019	Retrospective study	90	Rectum cancer (40), Colon-rectum cancer (21), Rectum injury (5), IBD (15), bladder cancer (7), Incontinence (2)	Laparoscopic modified keyhole technique	Colostomy (83), ileal conduit (7)	Unspecified	Parietex (88), Physiomesh (2)	Intraperitoneal	Seroma (4)	4 (2 with Physiomesh; 2 with Parietex)	12	The addition of some precautions to the laparoscopic KH technique makes it feasible and safe, with good results in terms of complications and rates of recurrence. The best results were associated with the use of Parietex compared to Physiomesh
S. Rajapandian [26]	2019	Retrospective study	23	Abdominoperineal resection (14), perineal trauma (3), and uncontrolled perineal sepsis (1); total proctocolectomy (2), carcinoma bladder (3)	Modified laparoscopic keyhole plus repair	Colostomy (18); ileostomy (2); ileal conduit (3)	0	Composite mesh	Intraperitoneal	Seroma (3)	23	1	The laparoscopic modified keyhole plus repair technique is a safe, feasible, and effective PH repair when performed by experienced surgeons. It has an accept- able recurrence rate and offers good cosmesis and functional outcomes
C. Bertoglio [37]	2020	Retrospective study	30	Miles (28), Harmann's (2), Cystoprostatectomy (2)	Keyhole (19), Sandwich Repair (13)	Colostomy (30), urostomy (2)	5	Gore-Tex Dual-Mesh (KH), DynaMesh-IPOM (SR)	Intraperitoneal	KH: parietal hematoma (1), Chronic seroma (2), bowel perforation (1). SB: chronic pain (1)	47	5 (KH); 0 (SR​)	Laparoscopic treatment of parastomal hernias with the “Sandwich Repair” technique seems to offer promising results, with a lower recurrence rate and less complications than the “Keyhole” technique
V. A. Gameza [24]	2020	Nonrandomized case-controlled prospective study	90	Cancer (51), IBD (43), Miscellaneous (41)	Keyhole technique (74), Sugarbaker technique (61)	Colostomy (90), ileostomy (41), urostomy (4)	40	Two-layer mesh of PPM and ePTFE (72), Coated lightweight mesh of polypropylene (63)	Intraperitoneal	Intraoperative lesion of bowel (keyhole 9 vs sugarbaker 5) Stomal outlet obstruction (3 vs 3) Stoma-cutaneous fistula (0 vs 1) Peristomal infection (2 vs 0) Intra-abdominal bleeding (1 vs 0) Peritonitis (3 vs 3)	57 (kH); 11 (SB)	Keyhole group (5) Sugarbaker group (6)	Study indicates that the Keyhole repair compares favorably with the Sugarbaker repair, provided a polypropylene mesh with an antiadhesive layer is used. With regard to early postoperative complications, recurrence, and late mesh-related morbidity, the Keyhole repair produces outcomes equal to the Sugarbaker technique
P. Keller [30]	2020	Retrospective study	62	Unspecified	Laparoscopic Sugarbaker repair (LPHR) or open (OPHR)	Colostomy (24)	Unspecified	Unspecified	Onlay, sublay, intraperitoneal	Wound complication (laparoscopic 9 vs open 16), Seroma/hematoma (8 vs 5), Superficial SSI (3 vs 10), Deep SSI (2 vs 7), Dehiscence (1 vs 9)	43 (LPHR), 12 (OPHR)	6 (LPHR), 20 (OPHR)	Laparoscopic repair of the parastomal hernia is associated with a shorter operating time, a reduction in the length of stay in the hospital, fewer short-term complications of wounds, and a longer service life than open repairs. Direct comparison of repair longevity between LPHR and OPHR with mesh using Kaplan–Meier estimation is unique to this study. Further studies are needed to better understand the methods of parastomal hernia repair associated with minor complications and increased duration
F. Tang [50]	2020	Prospective, observational study	16	Rectectomy (Mile surgery) (16), Colectomy (5), Radical cystectomy (1), Traumatic intestinal rupture (1)	Sugarbaker technique	Colostomy (16), Ileostomy (6), Ileal orthotopic neobladder (1)	11	Composite-polyester mesh (PCO-PM 20)	Intraperitoneal	Dyspnea (1), seroma (1), intestinal obstruction (2), urinary infection (2), wound infection (1)	24	0	PPP causes a significant increase in abdominal volumes preoperatively, thereby facilitating the total reintegration of the bowel into the abdominal cavity. Through the progressive increase of VAC, PPP induces respiratory adaptation to the elevat ed intra-abdominal pressure following hernia repair
A. G. Barranquero [36]	2023	Retrospective dual-center observational study	38	Sigmoidectomy (3), low anterior resection (12), abdominoperineal resection (21)	Sandwich technique	Loop colostomy (2), end colostomy (36)	11	TiMesh^®^ (29), DynaMesh^®^ IPOM (9)	Intraperitoneal	Seroma (15), Surgical site infection (2), Hematoma (3), Postoperative ileus (1)	39	3	The recurrence rates observed in the sandwich technique were in line with the rates documented in the current literature. Postoperative complications emerged as the primary risk factor for hernia recurrence in our study
J. Bellido-Luque [35]	2023	Prospective study	12	Abdominoperineal resection for rectal cancer (10); Hartmann (2)	Extraperitoneal modified Sugarbaker	End colostomy (12)	Unspecified	Optilene Mesh elastic	Retrorectus/left preperitoneal spaces	Subcutaneous emphysema (2); Seroma (2); partial bowel obstruction (1)	29 ± 5.7	0	This technique shows a low rate of intraoperative and postoperative complications with significant improvement in terms of pain and activities restriction compared to preoperatively
Robotic approach													
V. Maciel [16]	2018	Case report	2	Chronic fecal incontinence (1); low rectal cancer (1)	Retro-rectus robotic parastomal hernia	Colostomy (2)	0	Polypropylene macroporous mesh and a keyhole is created	Retromuscular plane	Obstructive symptoms (1)	12	0	This technique was successfully performed in two patients, without relapse after a year. The results suggest that this technique may offer an effective repair option, although it is more complex and time-consuming than other techniques such as laparoscopic Sugarbaker
S. A. Ayuso [17]	2020	Prospective study	4	Oncological (12) IBD (3)	Sugarbaker technique	Colostomy (4), ileostomy (6), urostomy (5)	0	Gore Dual-Mesh^®^ (13), Strattice™ (2)	Intraperitoneal	Pneumonia (1)	14.2 ± 9.4	1	This study describes a novel technique in which robotic parastomal hernia repair is performed with closure of the fascial defect. This study describes a technique that is safe, technically feasible, with low short-term hernia recurrence rates and low complication rates
M. Kyle [44]	2021	Retrospective study	16	Unspecified	Modified Sugarbaker technique	Ileostomy (7), colostomy (16), urostomy (1)	Unspecified	Synecor IP, gore BIO-A and Dual-Mesh	Intraperitoneal	Bowel obstruction (1), seroma (4), intestinal incarceration (1), colonic impingement (1), pneumonia (1), atrial fibrillation (2)	Unspecified	0	The robot-assisted repair of parastomal hernias is a reliable and reproducible procedure with few complications. Further long-term research is necessary to assess recurrence rates and potential late complications
J. R. Lambrecht [45]	2021	Prospective observational study	11	Abdomino-perineal resection for rectal cancer (11). anal incontinence (1), ulcerative colitis(1), constipation (1), urinary incontinence (1)	Endoscopic preperitoneal parastomal hernia repair (ePauli repair)	Colostomy (11), ileostomy (3), urostomy (1)	5	Dynamesh, uncoated synthetic mid-weight non-absorbable mesh with Bio-A synthetic absorbable mesh placed as barrier between the mesh and the bowel	Retromuscular plane	Serosa lesion (1), obstruction (2)	10	1	With our limited experience, we are encouraged with the pain, complication, and functional summary after ePauli repair and hopeful for the recurrence profle. ePauli/TAR is not for every patient or every surgeon and whether it should be restrained to recurrent PSH or be ofered as frst-line treatment for PSH is disputable
M. Dewulf [15]	2022	Prospective study	20	Rectal cancer (13), IBD, anal incontinence, anal fistula, constipation, diverticulitis	Pauli procedure	Colostomy (20), ileostomy (5), urostomy (1)	8	Large-pore, synthetic and nonabsorbable of polypropylene, or polyester material	Retromuscular plane	Serosa lesions (7), stoma necrosis (1), ileus (3), hematoma or seroma (4)	14	1	The Pauli technique was developed to decrease recurrence rates compared to key-hole retromuscular techniques, decrease surgical site occurrences compared to re-location techniques and to keep mesh away from the abdominal cavity

### Laparoscopic parastomal hernia repair

Laparoscopy has become over the last decade one of the approaches of choice in the surgical repair of PSH. The most employed laparoscopic techniques for PSH repair are the keyhole (KH) repair [19], the Sugarbaker (SB) technique [20, 21], and the sandwich technique [22–25]. Briefly, in the keyhole technique a mesh with a central hole or a slit allowing bowel to pass through the stoma site is positioned in an intraperitoneal fashion. Sugarbaker repairs consist in the intraperitoneal placement of a mesh to cover the stoma site after lateralizing the bowel, eventually after placement of sutures for primary closure of fascial defect to reduce the risk of recurrence [6, 26]. The sandwich technique is a combination of the KH and SB repair, using two intraperitoneal meshes. According to the original description [22], the first mesh is allocated in a KH fashion to stabilize the lateral abdominal wall in a perspective to reduce the risk of hernia recurrence in patients with a fascial defect lateral to the stoma. The second mesh is then positioned according to the SB principles accomplishing lateralization of the stoma loop. Considering these approaches as a cornerstone in PSH repair, modified techniques as well as interventions describing a combined open and laparoscopic approach have been proposed [10, 27–29], but limited to single center or even single surgeon experience.

Several studies have compared the laparoscopic approach with the open approach, showing how laparoscopy provides decreased rates of recurrence and postoperative complications compared to the open approach [6, 30]. Recently, Keller et al. [30] evaluated 62 consecutive patients who underwent either laparoscopic or open elective repair and compared the outcome of surgery. Each group consisted of 31 patients and had similar demographic details with a similar proportion of patients with colostomy. In the laparoscopic group, the same surgical repair was adopted in all patients. Open repair was performed more often on patients with prior PSH recurrence (58% vs 29%, p = 0.004). Compared to laparoscopic repair, patients who underwent open repair showed an increased incidence of wound dehiscence (29% vs 3%, p = 0.012), while non-wound complications were similar between the two groups. The operative duration (*p* < 0.001) and median length of in-hospital postoperative stay were shorter in the laparoscopic group (3 days vs 7 days, *p* < 0.001). After adjustment for prior repair, evaluation at 3 years reported a significantly longer recurrence-free time for laparoscopic repair than the open repair (*p* = 0.022).

Although relatively simple and easy to master, in the KH technique the presence of the central hole or of the slitting causes a weakness in the mesh which in turn increases the risk of PSH recurrence [6, 26]. The real risk of recurrence as well as the recurrence rate by adopting this technique has not been clearly assessed and remains controversial. As a matter of fact, an incidence of recurrence as high as 20.8% has been reported [29]. In the previous review on this topic it was stated that in laparoscopic repair, the SB technique was superior over the KH in terms of lesser rate of recurrence [8]. A recent nonrandomized case-controlled prospective study, comparing the outcome of patients operated on by a KH or an SB repair using a polypropylene mesh with an antiadhesive layer, showed an incidence of postoperative complications for the KH group of 14.9%, recurrence rate of 7% and late mesh-related morbidity of 8%. These results, with regard to end-colostomy PSH, did not differ significantly from those reported after an SB repair, in which postoperative complications were 11.5%, recurrence rate 10% and late mesh-related morbidity 10%. Therefore, KH repair produces outcomes somewhat similar, especially over the follow-up to the SB technique [24]. Similar results were obtained by Oma et al. [12]: in a cohort of 79 patients, the overall recurrence rate was 9%, without a significant difference in the risk of recurrence after KH compared to SB repair (1/10 = 10% vs 6/67 = 9%, p = 1.00).

Rajapandian et al. [26] looking for lesser and acceptable recurrence rates described the outcome of 23 patients, 18 of them with colostomy PSH, operated on with a modified KH repair. The technique consisted of an almost complete removal of the hernial sac to reduce the incidence of postoperative seroma, while to reduce the risk of recurrence the wall defect was approximated with a non-adsorbable suture, and the bowel fixed to the parietal wall with seromuscular sutures. The mesh was incised from one side to create a half slit to surround the bowel, with the cut margins overlapping each other and fixed with transfascial and intracorporeal sutures. No major intraoperative complications, such as bleeding or bowel injuries, or postoperative complications occurred. A further attempt in reducing recurrences through a modified KH repair was described by Olmi et al. [29] in a cohort of 90 patients, with 83 (92.2%) of them suffering an end-colostomy PSH and with a mean BMI of 30 kg/m^2^ (range 28–34). Repair was carried out through a reduction of the defect with multiple sutures with extracorporeal knotting, later covered and reinforced with a polypropylene mesh fixed with tacks. The stoma is fixed by suturing the edge of the defect and extracorporeal knotting, then the mesh with a slit of 3 cm in width for the stoma and an overlap of 5 cm is placed with the mesh border overlapped to create the KH. Finally, the mesh was fixed with a double crown of tacks. Postoperative complications consisted of seroma in four patients (4.4%), managed conservatively. Recurrence occurred in four patients (4.4%), in one case on postoperative day 7 due to technical error. Yan e al [10], in their series of 65 consecutive patients, 60 of them with colostomy, evaluated the effectiveness of combining an in situ re-ostomy with the laparoscopic KH technique. The main advantages of such an approach consisted in the opportunity to have an adequate operative space to dissect the bowel, closing the defect, stitching the mesh to the stomal bowel, and resecting the redundant bowel if necessary. Postoperative morbidity included two cases of seroma and three of ileus managed conservatively, while in one case of intestinal perforation a rescue surgery with intestinal resection and entero-enterostomy warranted care. Over a median follow-up of 29 months (range 3–60 months), no complications of mesh-related infection or patch erosion were noted, recurrence was recorded in one case (1.5%), 17 months after the index surgery.

Asif et al. [6] evaluated a cohort of 49 patients with PSH without any differences in terms of demographic features. SB repair was conducted in 14 and compared with 19 laparoscopic KH, 11 re-siting, and 5 open repairs. There were no differences in terms of postoperative complications among all techniques, but the SB group showed a significantly lower rate of PSH recurrence. Hansson et al. [31] described the outcome of 61 consecutive patients, 55 with symptomatic colostomy-related PSH who underwent laparoscopic SB technique with positioning of an ePTFE mesh (Gore-Tex Dual-Mesh Biomaterial^(R)^, WL Gore Associates Newark, DE, USA). The morbidity rate was 19%, and one patient died due to metastasis of lung carcinoma causing a bowel obstruction. The recurrence rate was 6.6% after a mean follow-up of 26 months. DeAsis et al. [23] compared the outcome of 25 SB compared to 37 other PSH repairs including 18 KH, 13 re-siting, and 6 open repairs in a homogeneous group of 62 patients suffering from PSH. The authors proposed a modified SB technique consisting of a primary closure of the wall defect by a combined transfascial and intracorporal suture before placing the mesh. Patients who had SB repair experienced lesser postoperative complication rate (40% vs 76%, p = 0.02) as well as reduced recurrence rate (16% vs 60% p < 0.001), so showing a protective role against recurrence. Although rarely reported [32, 33], to avoid the risk of mesh infection, obstruction, fistulization or mesh erosion, Rege et al. [34] proposed a modified mesh placement technique. A composite mesh was placed with upward folding of the visceral non-adhesive surface in contact with the stomal loop with the aim of avoiding the risk of mesh erosion into the bowel or causing adhesions and subsequent stoma obstruction. In the reported series of 14 patients, over a follow-up ranging between 15 months and 7 years, no complications of mesh such as seroma formation, stomal necrosis, mesh infections and erosion, or hernia recurrence were noted. To overcome the risk of adhesions and fistula formation in the report of Bellido-Luque [35], a modified SB approach based on an extended totally extraperitoneal mesh repair was proposed. The authors described a series of 12 patients who underwent an end-colostomy PSH repair with an average follow-up of 29 months. The proposed technique where the mesh does not need helical sutures allowed a significant improvement in pain and activity restriction compared to preoperatively, no postoperative wound infections or hematomas were noted, and in two cases (16%) a seroma was detected and managed conservatively. One patient (8%) required postoperative readmission (within 1 month after the index surgery) due to partial bowel obstruction without the need of reintervention. No follow-up dropout or recurrence was recorded in the whole series [35].

By adopting the sandwich technique, in the report by Barranquero et al. [36], repair was performed in 38 patients, with a median BMI of 29.2 kg/m^2^ whose hernia was associated with an end colostomy in 94.7% of cases. The recurrence rate was 7.9%, with a median time for recurrence of 12 months, and a median follow-up of 39 months. Also, diagnosis was made in more than 92% of patients by CT scan examination. Postoperative complications were found in the analysis as the main risk factor for hernia recurrence. Bertoglio et al. [37] compared the outcomes after repair between 13 patients who underwent sandwich technique and 19 patients operated on with a KH repair. Patients’ demographics, characteristic of the defect, and postoperative outcomes were similar between the two groups, while in the sandwich repair group a statistically significant shorter length of hospital stay (p = 0.04) compared to KH repair was noted. He recurrence rate was 21% in the KH group at the 1-year follow-up, while no overall recurrences were recorded in the sandwich group over a median follow-up of 26 months (range 13–78). Some other techniques have been reported in the literature. Fischer et al. [27] described a technique of PSH repair with a 3D funnel-shaped intraperitoneal mesh device and same-sided stoma relocation. The authors reported the results in 56 patients; end-colostomy PSH accounted for 84% and the repair was attempted by laparoscopy in 41 (73%) patients. The median follow-up time was 38 months (range 12–58 months) and over this time, if the overall recurrence rate was 12.5% (7/56), the recurrence rate in the laparoscopic group accounted for 7.3% (3/41), while in the open group it was 26.7% (4/15). The overall incidence of surgical complications was 16.7% (9/56), with major surgical complication rate of 8.9% (5/56). Szczepkowski et al. [28] described an alternative approach called hyper/SPHR technique (hybrid parastomal endoscopic re-do/Szczepkowski parastomal hernia repair). This technique consists of four steps as a combination of laparoscopic and open approach (laparoscopic stage, open stage, reconversion to laparoscopy, and final open stage with neo-stoma formation) with the use of a 3D pre-shaped mesh placed intraperitoneally with a hole in which the ostomy bowel is delivered and the funnel oriented to the visceral side of the abdomen. In this series, 12 patients with end-colostomy PSH were operated on, and a single (8.3%) postoperative complication (a small wound hematoma treated conservatively) was detected. Also, no stoma site infections, stoma-related problems, or recurrence were recorded over a 13.5-month (range 6–17 months) follow-up. A combined laparoscopic and ostomy-opening approach was also proposed by Kohler et al. [38]. The repair was based on the placement of a cylindrical-shaped mesh of 4 cm funnel length after laparoscopic adhesiolysis and an open approach with excision of the ostomy opening and closure of the bowel later delivered through the funnel of the mesh. The mesh is inserted in the peritoneal cavity through the hernia defect. Laparoscopy is then restored after suture to close the defect to fix the mesh to the peritoneum. No mesh complications were reported or recurrence noted over a 4-month follow-up (range 3–8 months). Although these are promising results, to the best of our knowledge, up-to-date results derived from a wide validation of these repairs are lacking.

### Robotic parastomal hernia repair

Robotic surgery has been integrated into the surgical landscape for over 20 years and has particularly experienced rapid growth in the field of general surgery, in an effort to overcome the limitations that laparoscopy, and even more open surgical techniques, may impose on surgeons.

Preliminary series report encouraging results, although not yet validated by randomized controlled trials (RCTs), especially in the short term, when a robotic approach is employed in ventral hernia repair, [39–43], and similarly happens when a robotic repair of PSH is evaluated. The present literature review has shown the paucity of these primary studies, and our aim is therefore to provide an overview of the current state of the art and to guide potential primary studies in this direction. Of the articles included in the review, five studies were analyzed for robotic repair of PSH [15–17, 44, 45]. Four are retrospective studies, one is a prospective study, and included in total 61 patients with a median follow-up of 1 year.

Kyle et al. [44] conducted a retrospective study on the postoperative outcomes for a total of 24 patients, of whom 16 underwent colostomies treated with a modified robotic Sugarbaker technique. In any case was necessary conversion to a laparoscopic or open procedure. Two patients underwent reoperation, one during hospitalization for intestinal obstruction and the other later for the development of a hernia between the mesh used for incisional hernia repair and the mesh placed for parastomal hernia. 33% of patients had a minor complication, and there was a 16% incidence of seroma. The authors clarified that the two major complications occurred in the early period when the learning curve for using the new robotic platform was not yet optimal. With more frequent use of the platform, both the operative time and docking time were reduced. Dewulf et al. [15] presented preliminary results in a retrospective study on 62 patients: 38 of whom were operated on for end-colostomy PSH parastomal hernia. Technical considerations of robot-assisted modified Sugarbaker repair, robot-assisted Pauli technique, and minimally invasive use of a funnel-shaped mesh in the treatment of parastomal hernias were evaluated. Patients who underwent robotic modified Sugarbaker procedure had an average hospital stay of 2 days, an operative time of about 180 min, and there was no need for hospital readmission within 30 days postoperatively. One patient developed a recurrence, with an average follow-up of 14 months. Using the Pauli technique, transversus abdominis release (TAR) combined with the advantages of visceral parietalization, serosa injuries occurred in seven patients, and in one mucocutaneous detachment led to stoma revision, with an average operation time of 156 min, a medium hospital stay of 3 days, and mean follow-up of 14 months. There were minor postoperative complications in eight patients, one patient had a recurrence, and one patient had stoma necrosis. For patients who received a funnel-shaped mesh, longer mean operative times was noted, 201 min, but the authors state that the funnel configuration facilitates stoma irrigation, which could otherwise be compromised by the lateralization of the intestine after the modified Sugarbaker and Pauli procedures. Lambrecht [45] included in his prospective observational study 15 patients with parastomal hernia who underwent a repair procedure, termed ePauli with transversus abdominis release (TAR), 9 of whom were approached robotically. The experience, although limited and with an average follow-up of only 10 months, has shown encouraging results in terms of complications, feasibility, and recurrence. The surgical technique used was technically challenging, but feasible, safer, and less strenuous when performed robotically, and more suitable for patients with recurrent parastomal hernia who had previous hernia repairs with intraperitoneal or retromuscular mesh.

Ayuso et al. [17] retrospectively analyzed the demographic data and monitored intraoperative and postoperative outcomes for 15 patients operated on by robotic Sugarbaker technique for parastomal hernia repair, of which 4 had an end colostomy. The average operative time was 182 min, and there was only one recurrence observed over an average follow-up period of 14.2 months. In conclusion, the robotic Sugarbaker technique has proven to be safe and technically feasible, with low rates of short-term recurrence and minor complications. Another robotic technique reported in the literature is analyzed by Maciel et al. [16], who presented data obtained from a retrospective analysis of two patients who underwent robotic retromuscular parastomal hernia repair, in both cases involving colostomy and midline incisional repair. The follow-up period was 1 year for both patients, and no recurrences were observed. According to the authors, in agreement with other studies included in the review, the technique is certainly challenging and requires a high level of surgical expertise with abdominal wall dissection techniques, and a longer operative time compared to laparoscopic techniques.

## Discussion

This systematic review, after the former manuscript by Hansson et al. [8], critically analyzed the evolution of surgical treatment with specific regard to the up-to-date efficacy of mininvasive techniques in PSH repair. Parastomal hernia prevention and treatment are a priority in colorectal pathology, whose incidence as well as recurrence rate, based on the current evidence, is far to be perfectly known with repair as a challenge for surgeons. If the accuracy of the detection rate is affected by the diagnostic method employed, the recurrence rate is strongly dependent on the follow-up time. The risk of recurrence potentially might increase over time, and a minimum follow-up of at least 5 years has been advocated to evaluate the effectiveness of surgical repair [46].

Over the past decade, laparoscopy has emerged as one of the preferred approaches due to its various advantages, including decreased postoperative pain, shorter hospital stays, and lower complication rates, [8, 26, 31]. A significant shift from open to laparoscopic repair methods has been observed, reflecting advancements in surgical techniques and patient outcomes. Keller et al. [30] provided pivotal comparative data, indicating that laparoscopic repair not only reduces the length of in-hospital stay, but also significantly lowers recurrence rates compared to open surgery. This aligns with the findings of Asif [6] and Keller [30], underscoring the procedural benefits of laparoscopy.

Although widely used, the keyhole, the Sugarbaker, and the other repair techniques described have not definitively shown which surgical technique should be chosen; hopefully, ongoing randomized controlled trial will clarify this issue [5]. The KH technique, despite its simplicity, presents a concern for increased recurrence risk due to the inherent mesh weakness [6, 26]. Contrarily, the SB technique, as elucidated by Sugarbaker [20, 21], and the sandwich technique have shown promising results in reducing recurrence rates. However, the optimal choice among these techniques remains a subject of ongoing research and debate.

Rajapandian et al. [26] and Olmi et al. [29] have contributed to refining the KH technique, focusing on reducing recurrence risks through modified surgical procedures. Their efforts in minimizing postoperative complications, such as seroma formation, have been noteworthy. Similarly, Sugarbaker's technique has been modified by various researchers [23, 34] to reduce mesh-related complications, further enhancing the safety profile of laparoscopic PSH repairs.

If the advantages of a mininvasive approach compared to an open approach in terms of postoperative morbidity are well recognized, what defines the technique to be adopted to warrant better long-term results is yet unclear. Surgical repair of PSH might be considered an evolving paradigm and an unsolved issue. If SB technique was previously considered to be the repair of choice because of the evidence of fewer rates of recurrence [8], this has been rebutted in recent studies where comparable results in terms of both perioperative complications and recurrence rate were reported [24]. This evidence might reflect a deep influence of surgeons’ expertise and attitude over PSH diagnosis and repair. As a matter of fact, despite the continuous research of modification in the technique aimed to ameliorate short- and long-term outcomes, the heterogeneity of patients without definitive and standardized criteria in defining patient selection, PSH characteristics, surgical techniques and even mesh selection could all together raise major confounding factors when analyzing data. The current literature in fact focuses on the ability of surgeons to modify the available technique as well as in suggesting new ones and introducing more and more meshes with specific features. In this spirit, the sandwich technique was introduced. Such an approach is a blend of the KH and SB methods. The studies reported by Barranquero et al. [36] and Bertoglio et al. [37] showed a lower recurrence rate and shorter in-hospital stay when patients were operated on with such an approach, so suggesting a potential advantage over singular techniques.

Moreover, innovative approaches such as the hyper/SPHR technique [28] and the combined laparoscopic and ostomy-opening approach [38] have expanded the scope of laparoscopic PSH repair, showing promise in reducing postoperative complications and recurrence rates.

In conclusion, the shift toward laparoscopic techniques for PSH repair has shown significant benefits in terms of reduced postoperative complications, shorter in-hospital stays, and lower recurrence rates. While the KH, SB, and sandwich techniques remain the most commonly employed, ongoing refinements and innovations continue to enhance their efficacy and safety. Surgeons’ expertise, preferences, and attitudes when approaching PSH more than the technique used in itself for the PSH repair might influence deeply the outcome. This might partly explain the similarities in the outcome reported in more recent studies. Standardization in future research is mandatory and should focus on long-term outcomes and direct comparisons between the available techniques to establish clear guidelines for optimal surgical intervention in PSH repair.

The surgical treatment of PSH has evolved significantly over the years, especially with the advent of robotic-assisted techniques. Initially, PSH was managed with relocation techniques or local suture repairs, but these approaches often resulted in high recurrence rates and complications [15, 44]. The introduction of mesh-based techniques marked a significant advancement, and these have been further refined with the development of robotic-assisted surgery. The complexity and challenges associated with PSH repair are well documented. The robotic approach to PSH repair has specific technical requirements. Patient positioning and trocar placement are crucial for optimizing the use of robotic arms and ensuring adequate exposure and working space. Various types of mesh are used depending on the hernia and patient condition, with careful consideration of potential complications from the mesh material. The robotic system offers enhanced visualization and precision, which can be particularly beneficial in complex dissections and mesh placements. In the hands of an experienced surgeon, the initial stages of the learning curve have shown an increase in operative times and complications [15, 16, 44]. According to the study by Kyle et al. [44], it is evident, even with preliminary data, that since the surgical treatment of parastomal hernia is complex, prone to complications and recurrences, and given the variety of minimally invasive techniques available without a defined preference for one over the other, particularly for robotic approach techniques, it is essential for surgeons to have a learning curve that can improve the surgical technique. Complications, in fact, occurred in the early phases, and further research is needed to provide more evidence on what the optimal surgical technique might be. The limitation of the study is the absence of long-term follow-up, which would provide more precise indications on the recurrence rate, not evaluated in this case. Among the analyzed outcomes, an acceptable recurrence rate was noted: out of 82 patients, 53 with colostomies, 3 experienced a recurrence during follow-up, accounting for 3.65%. Although this figure still needs to be confirmed, it is certainly acceptable, demonstrating that the robotic technique is effective. However, to definitively label it as such, this finding will need to be confirmed by further primary studies. Observational studies comparing laparoscopic and robotic end-colostomy PSH are needed in the literature. The findings from our systematic review regarding the minimally invasive robotic approach are based on a limited number of studies; therefore, our goal is to promote and guide scientific research in this direction. The EHS guidelines (year 2018) [47] recommend the use of a mesh without hole for laparoscopic repair, as with the Sugarbaker method in preference to a keyhole approach, but this assumption might need a revision because of the current uncertainty on the real superiority of such a technique over the KH, so that further research in this area is mandatory. The absence of strong recommendations reflects the general paucity of high-quality evidence to guide the choice of technique and materials for the repair of PSHs, both laparoscopic and even more so for robotic repair techniques, emphasizing, as we do, the need for additional comparative studies to better inform clinical practice.

Nevertheless, this review has several limitations, since it is prevalently limited to observational, retrospective and nonrandomized studies often describing a small series of patients and often without a clear distinction between end colostomy and other kinds of PSH, which makes it difficult to evaluate the reported data and so obtain a clear indication. As a matter of fact, the evaluation of the studies included highlighted several biases regarding the diagnostic criteria for hernia recurrence, description of patient features, and hernia-related risk factors, as well as the evidence of a direct correlation between surgeon expertise and results reported. The heterogeneity depicted in each of these parameters does not allow to define the superiority of a specific approach over others, because further primary, multicentric, and larger cohort studies with homogeneous patients’ criteria selection, standardized surgical technique, and accurate data collection with an up to 5 years follow-up are needed.

## Conclusion

PSH repair is an unresolved and challenging issue for general, colorectal, and hernia surgeons, reason because rightly falls within the definition of a complex abdomen. Nowadays, minimally invasive surgical techniques have shown their effectiveness in the management of this pathology, yet among the various techniques described for laparoscopic or robotic approaches, which treatment to choose has not been clarified. This manuscript, despite the limitations, provides a state-of-art overview on both robotic and laparoscopic strategies in the surgical treatment of PSH and could be of inspiration for further evaluation. Hopefully, well-designed and large cohort series randomized controlled trials will provide definitive and widely accepted indications on the laparoscopic or robotic technique to choose when approaching a PSH repair, which in turn might be eventually useful in a revision of the EHS guidelines as well. Accurate patients’ selection, PSH definition, and the choice of the mesh will be of paramount importance. Of course, an adequate long-term follow-up would be necessary to define the real rate of recurrence.

## Appendix

("robot"[All Fields] OR "robot s"[All Fields] OR "robotically"[All Fields] OR "robotics"[MeSH Terms] OR "robotics"[All Fields] OR "robotic"[All Fields] OR "robotization"[All Fields] OR "robotized"[All Fields] OR "robots"[All Fields] OR ("laparoscopes"[MeSH Terms] OR "laparoscopes"[All Fields] OR "laparoscope"[All Fields] OR "laparoscopical"[All Fields] OR "laparoscopically"[All Fields] OR "laparoscopics"[All Fields] OR "laparoscopy"[MeSH Terms] OR "laparoscopy"[All Fields] OR "laparoscopic"[All Fields])) AND ("repairability"[All Fields] OR "repairable"[All Fields] OR "repaire"[All Fields] OR "repaired"[All Fields] OR "repairment"[All Fields] OR "wound healing"[MeSH Terms] OR ("wound"[All Fields] AND "healing"[All Fields]) OR "wound healing"[All Fields] OR "repair"[All Fields] OR "repairing"[All Fields] OR "repairs"[All Fields]) AND (("colostomy"[MeSH Terms] OR "colostomy"[All Fields] OR "colostomies"[All Fields] OR ("hartmann"[All Fields] OR "hartmann s"[All Fields] OR "hartmanns"[All Fields]) OR ("ostomy"[MeSH Terms] OR "ostomy"[All Fields] OR "ostomies"[All Fields])) AND ("parastomal"[All Fields] AND ("hernia"[MeSH Terms] OR "hernia"[All Fields] OR "hernias"[All Fields] OR "hernia s"[All Fields] OR "herniae"[All Fields]))).
